# Rapid Multiple Immunoenzyme Assay of Mycotoxins

**DOI:** 10.3390/toxins7020238

**Published:** 2015-01-27

**Authors:** Alexandr E. Urusov, Anatoly V. Zherdev, Alina V. Petrakova, Elchin G. Sadykhov, Olga V. Koroleva, Boris B. Dzantiev

**Affiliations:** A.N. Bach Institute of Biochemistry of the Russian Academy of Sciences, Leninsky Prospect 33, 119071 Moscow, Russia; E-Mails: urusov.alexandr@gmail.com (A.E.U.); zherdev@inbi.ras.ru (A.V.Z.); alina.petrakova@gmail.com (A.V.P.); elchins@gmail.com (E.G.S.); koroleva@inbi.ras.ru (O.V.K.)

**Keywords:** mycotoxins, zearalenone, aflatoxin B1, ochratoxin A, ELISA, kinetic immunoassay, biotin-streptavidin reaction, methanol extracts

## Abstract

Mycotoxins are low molecular weight fungal metabolites that pose a threat as toxic contaminants of food products, thereby necessitating their effective monitoring and control. Microplate ELISA can be used for this purpose, but this method is characteristically time consuming, with a duration extending to several hours. This report proposes a variant of the ELISA method for the detection and quantification of three mycotoxins, ochratoxin A, aflatoxin B1 and zearalenone, in the kinetic regime. The main requirement for the proposed kinetic protocol was to provide a rapid method that combined sensitivity and accuracy. The use of biotin with an extended spacer together with a streptavidin–polyperoxidase conjugate provided high signal levels, despite these interactions occurring under non-equilibrium conditions. Duration of the individual mycotoxin assays was 20 min, whereas the analysis of all three mycotoxins in parallel reached a maximum duration of 25 min. Recovery of at least 95% mycotoxins in water-organic extracts was shown. The developed assays were successfully validated using poultry processing products and corn samples spiked with known quantities of mycotoxins. The detection limits for aflatoxin B1, ochratoxin A and zearalenone in these substances were 0.24, 1.2 and 3 ng/g, respectively.

## 1. Introduction

The term “mycotoxin” incorporates all the various secondary metabolites of molds. Although the negative effects of mycotoxins have been described since the middle ages (15–16th centuries [1]) scientists only began to engage in the identification and monitoring of specific hazardous compounds in the from the mid-20th century. To date, more than 500 mycotoxins are known, but only a few dozen of these may be found in food and animal feed in potentially dangerous quantities [2]. These toxins are produced mainly by five genera of fungi: *Aspergillus*, *Penicillium*, *Fusarium*, *Alternaria* and *Claviceps* [3]. Most mycotoxins are very stable with respect to temperature and chemical exposure. Plant products are contaminated by mycotoxins directly as growing crops, whereas animal products contain mycotoxins assimilated from contaminated feeds. The complex toxic effects of mycotoxins pose a significant risk to human and animal health and necessitate their effective monitoring and control [4,5].

Currently, most countries around the world have established regulatory requirements for the maximal permissible residue levels (MPRLs) of mycotoxins in various foods and feed [4,6]. Because there are considerable variations in the degree of contamination and the probability of mycotoxins being present in concentrations not exceeding MPRLs, accurate and sensitive quantitative detection methods are of primary importance. The development of such methods is mainly focused on chromatography [7,8], chromatography coupled with mass spectrometry [9,10,11,12,13] or immunoassays [14,15,16,17]. Chromatography and mass spectrometry require the use of complex and expensive equipment and are therefore mainly applied for confirmatory analysis. Immunoassay methods, among which the enzyme-linked immunosorbent assay (ELISA) is the most commonly used format [18,19], are much simpler in design, implemented using relatively inexpensive equipment, provide high productivity by allowing the simultaneous testing of tens of samples, and enable the detection of mycotoxins with high sensitivity and accuracy [20].

However, common ELISA kits require that all immunochemical interactions take place at equilibrium (or close to equilibrium), thus providing good reproducibility. As a result, total assay duration for different kits varies from one hour to several hours. For example, the application of Aflatoxins B1 [AFB1] ELISA Test Kit (Krishgen Biosystems, Los Angeles, CA, USA) requires 60 min, Zearalenone (ZEN) ELISA Kit (Cusabio Biotech Co., Ltd., Wuhan, China)—70 min, IDetect Ochratoxin A ELISA Test Kit (Idlabs Biotechnology Inc., London, ON, Canada)—75 min, Total Aflatoxin ELISA Kit (EuroClone SpA, Milan, Italy)—80 min, Aflatoxins B1 in food kit (Diagnostic Automation, Inc., Calabasas, CA, USA)—150 min.

This method also requires the transition of ELISA to the kinetic regime, and this is associated with a decrease in the number of immune complexes generated and detected, and accordingly, produces less accurate assay results. For certain applications though, assay durations of several hours are often unacceptable. Many tasks involved in raw material control, process monitoring and final product testing require a more rapid testing turnaround time. However, known methods of rapid immunoassay such as immunochromatography are focused primarily on qualitative “yes–no” testing and cannot serve as an adequate substitute for ELISA.

The kinetics of the interactions in ELISA have been studied in a number of works, including both theoretical research, starting with the classic papers of Rodbard [21,22], and experimental research [23,24,25,26,27]. However, the application of this knowledge towards the development of express immunotechniques is very limited. The shortening of the analysis duration to 10–50 min [28,29] has been described only for specific individual antigens based on empirical evaluation of the binding kinetics. In addition, a shift to the kinetic regime reduces (to a greater or lesser degree) the binding of detectable markers, which must be accompanied by additional solutions to retain acceptable accuracy of analysis results. Methodological solutions for express immunoassays are provided in commercial tests (see www.neogen.com/foodsafety/fs_da_index.html as an example), but the underlying methods are not disclosed by manufacturers. Therefore, the question of the simultaneous control of several compounds in the kinetic regime, which is especially important for the monitoring of mycotoxin contamination, remains open. It remains unclear to what extent the kinetics of immunochemical interactions vary for different antigens, and whether their combination in a single assay protocol is possible.

Given the above, our aim was to study the possibility of rapid control of multiple mycotoxins using a unified protocol based on modified microplate ELISA. The unification of stages duration allows to carry out all floods/incubations/washings simultaneously in different wells of microplate containing immunoreactants of different specificity and by this way to obtain information about all controlled mycotoxins after one assay cycle. Three mycotoxins, aflatoxin B1 (AFB1), ochratoxin A (OTA), and zearalenone (ZEA), were selected as test compounds owing to their wide presence in food stuffs and the significant threat they pose to consumers [10,14,15]. Efficiency of the developed assay was validated using corn samples as one from priority foodstuffs contaminated by mycotoxins [30] and poultry processing samples due to importance of contamination control along food chain and a lack of corresponding investigations for different matrixes of animal origin [31,32].

## 2. Results and Discussion

### 2.1. Choice of Assay Format and Overall Optimization

Two approaches to analyte labeling are generally used in ELISA, namely direct and indirect labeling [33]. The first proposes the use of direct complexes between the enzyme and specific antibodies or competing antigen, thus reducing the number of incubation steps in the course of the assay. However, this approach can be influenced by negative effects of the test sample matrix on the enzyme label and the risk of enzyme inactivation. In the second approach, the enzyme is introduced to the assay only once the immune complexes have been formed. This approach eliminates contact between the sample matrix and the label and allows the use of the same labeled reagent for multiple analytes. In the case of mycotoxins, organic solvents and a wide range of extracted substances may inactivate the enzyme label. We therefore chose the indirect labeling of antibodies for our assay.

The proposed kinetic ELISA was accomplished with the use of several additional reagents or steps, thereby enabling the effective incorporation of the label in the complexes to be detected: (1) the biotin-streptavidin interaction was exploited for the detection of primary antibodies, as this complex has a higher binding constant (10^15^ M^−1^) compared with the interaction between primary antibodies and anti-species antibodies [34]; (2) specific primary antibodies were modified with a biotin ester containing a 14-atom spacer (biotinamidohexanoyl-6-aminohexanoic acid *N*-hydroxysuccinimide ester) [35,36]; and (3) the streptavidin moiety was modified with a polymer that enabled the coupling of several peroxidase molecules [37,38].

**Figure 1:**
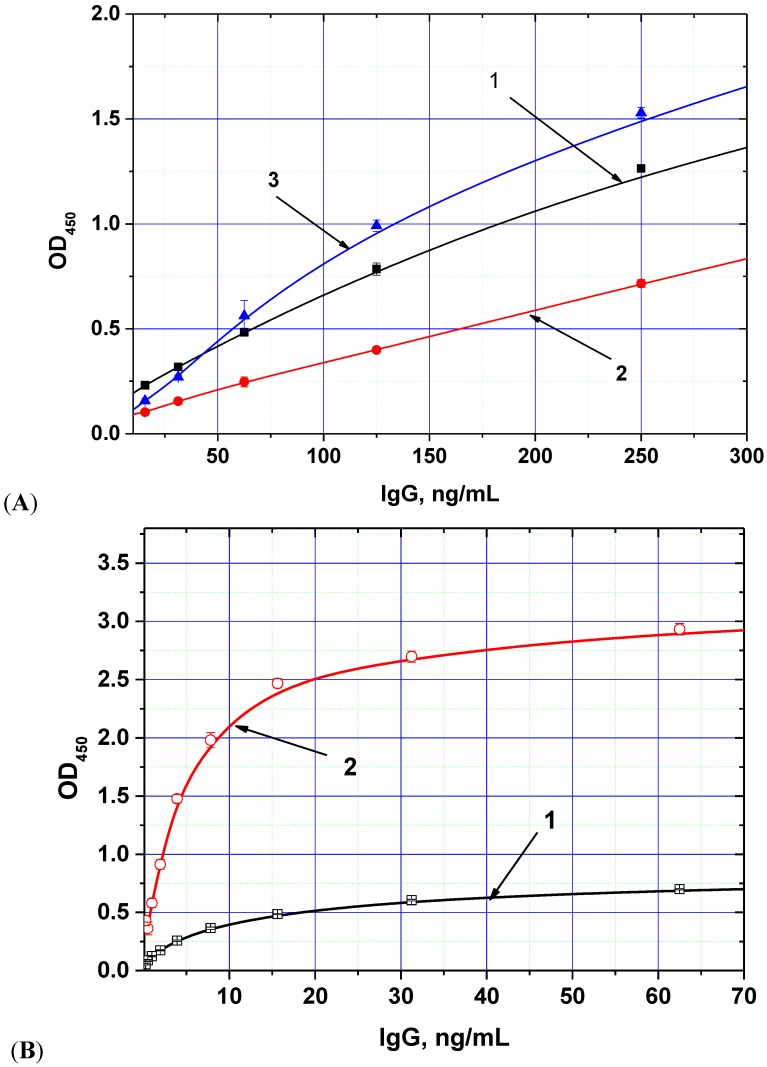
Dependences of optical density (OD) registered in the ELISA from the concentration of anti-AFB1 antibodies obtained for different methods of immune complex labeling. (**A**) variants of peroxidase conjugate binding (1—antibody-biotin + antispecies antibody—peroxidase, 2—antibody-biotin + streptavidin-peroxidase, 3—antibody-additional bridge-biotin + streptavidin-peroxidase); (**B**) variants of peroxidase conjugates (1—antispecies antibody—peroxidase, 2—streptavidin-polyperoxidase). AFB1-BSA conjugate was immobilized in microplate wells from 0.5 μg/mL; other parameters of the ELISA experiment are given in the Experimental Section (data are presented for three replicates).

The chosen biotin derivative demonstrated two-fold increased binding to the labeled streptavidin compared with the usual biotin *N*-hydroxysuccinimide ester (see Figure 1A), whereas the streptavidin–polyperoxidase conjugate provided up to 5-fold increase of detected peroxidase activity (Figure 1B).

**Figure 2:**
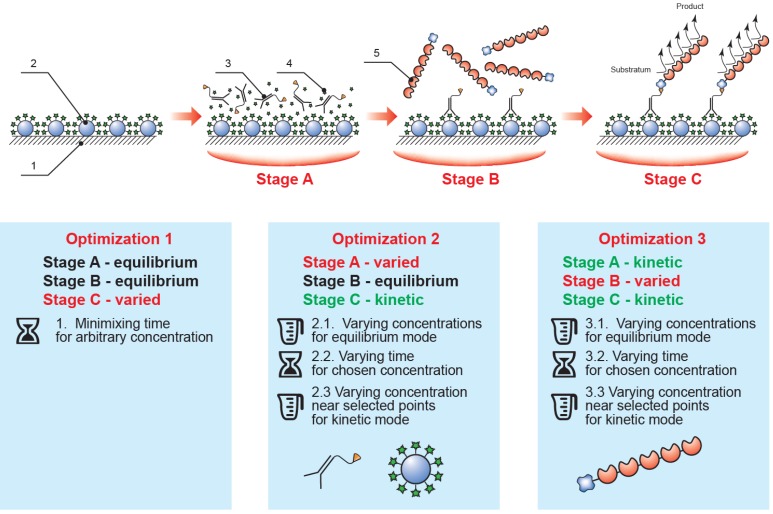
The proposed rapid ELISA format and the optimization sequence employed (1, polystyrene microplate; 2, immobilized mycotoxin–protein conjugate; 3, specific antibody–biotin conjugate; 4, mycotoxin in the tested sample; 5, streptavidin–polyperoxidase conjugate; stage A, competitive immunochemical interaction; stage B, interaction of specific complexes with streptavidin-polyperoxidase; stage C, enzymatic reaction).

Thus, the implemented analysis included three sequential steps (see Figure 2): (1) competitive interaction of the biotinylated antibody with immobilized mycotoxin–protein conjugate and free mycotoxin potentially contained in the sample; (2) interaction of the streptavidin–polyperoxidase conjugate with the biotin moiety of the formed immune complexes; and (3) detection of the catalytic activity of the bound enzyme label. Between each step the microplate was washed to remove any unbound components. The ELISA calibration curve was therefore determined by the conditions of the competitive interaction step (1), with the subsequent steps (2–3) merely allowing identification of complexes formed in the first step. This approach facilitates the manipulation of the parameters of the various stages to optimize them.

Development and optimization of the assay protocol was initiated from equilibrium conditions: steps (1) and (2), duration of 60 min; step (3) duration of 20 min. We confirmed experimentally that any further increase in step duration did not lead to a significant increase in the signal amplitude of the ELISA, OD_max_ (which refers to the optical density at 450 nm recorded after carrying out the ELISA in the absence of target analyte in the sample).

Next, the enzymatic step was optimized. We selected saturating concentrations of the immobilized mycotoxin–protein conjugate, specific biotinylated antibodies and the streptavidin–polyperoxidase conjugate. The final step duration was chosen to provide the achievement of OD_max_ in the range of 0.5–1.0, which permits quantitative determination of the analyte with maximum accuracy [39].

Following optimization, the concentrations of the reactants and the step durations were consistently set for steps (1) and (2), whereas for step (3), the OD_max_ values were controlled so they would remain within the optimal range of 0.5–1.0 to reach the lowest possible limit of analyte detection. To provide ELISA-like accuracy, the resulting OD_max_ after all optimizations should not fall below 0.5.

### 2.2. Reducing the Duration of the Enzymatic Reaction

In accordance with the above principles, the development of the rapid ELISA method was then initiated for the assay of the three mycotoxins, AFB1, OTA, and ZEA, under equilibrium mode. Analytical characteristics of the corresponding protocols are shown in Table 1.

**Table 1:** Analytical characteristics of mycotoxin ELISAs under equilibrium mode.

Mycotoxin	Analysis time, min	Limit of detection, ng/mL	Working range, ng/mL	Maximum deviation *, %	Average deviation *, %
AFB1	140	0.1	0.1–1.0	12.3	3.4
OTA		4.0	4.0–120.0	11.6	4.2
ZEA		0.3	0.3–50.0	14.2	4.7

* Measurements were carried out using four concentrations within the working range, *n* = 4.

The duration of the enzymatic reaction was optimized as described above. The resulting experimental data (Table 2) demonstrated that an 8 min incubation period was sufficient for ELISA of all three mycotoxins.

**Table 2:** Dependence of ELISA signal amplitude (OD_max_) on the duration of the enzymatic reaction.

Mycotoxins	Time, min			
	5	8	12	15
AFB1	0.32	0.64	0.82	0.89
ОТА	0.27	0.58	0.76	0.93
ZEA	0.44	0.73	0.92	0.87

### 2.3. Choice of Reagent Concentrations for Competitive Immune Interaction

Considering the first step of the assay, we initially studied the most efficient immobilization procedure for the mycotoxin conjugates. The essential issue was the buffer choice for immobilization. For this purpose, the common recommendations for ELISA propose sodium-carbonate buffers of varying ionic strength with a pH of 9.0–9.5 [39]. However, this is based on data obtained using antibodies, and is therefore not automatically valid for other proteins that may have different isoelectric points, hydrophobicity, molecular weights, and other characteristics. Initial comparisons of the competitive curves obtained by immobilization of the conjugates in phosphate (pH 7.4) and carbonate (pH 9.5) buffers showed negligible differences in terms of signal amplitude and reproducibility (data not shown). The use of the same media for initial immobilization and subsequent incubations was also considered preferable because this minimizes the risk of desorption and/or conformational changes of the adsorbed proteins. For this reason, in our study, microplate preparation was carried out using 50 mM phosphate buffer, pH 7.4, containing 100 mM NaCl (PBS).

**Figure 3:**
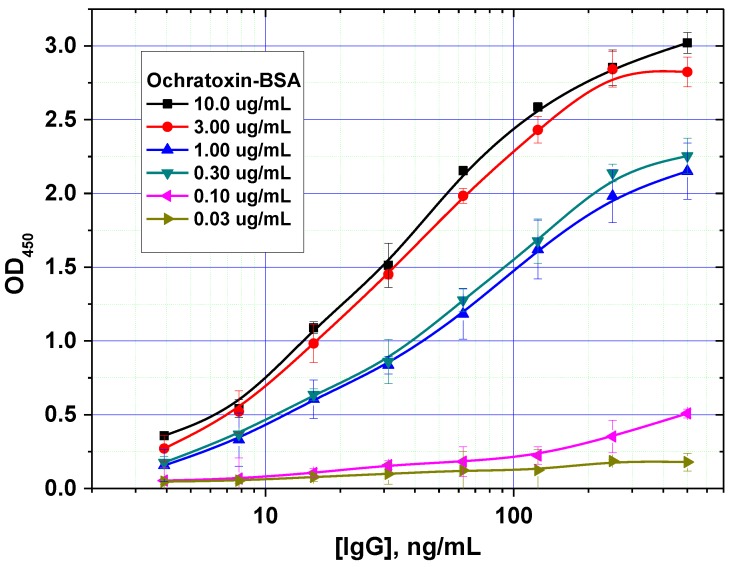
Optimization of the concentration of immobilized ochratoxin A–bovine serum albumin (OTA–BSA) conjugate (data are presented for three replicates).

In addition, the optimal concentration of the immobilized mycotoxin–protein conjugate was determined. An example of such a comparison is shown in Figure 3 for OTA. For this mycotoxin, a concentration of 0.3 µg/mL was shown to achieve the minimal limit of detection without loss of assay accuracy.

**Figure 4:**
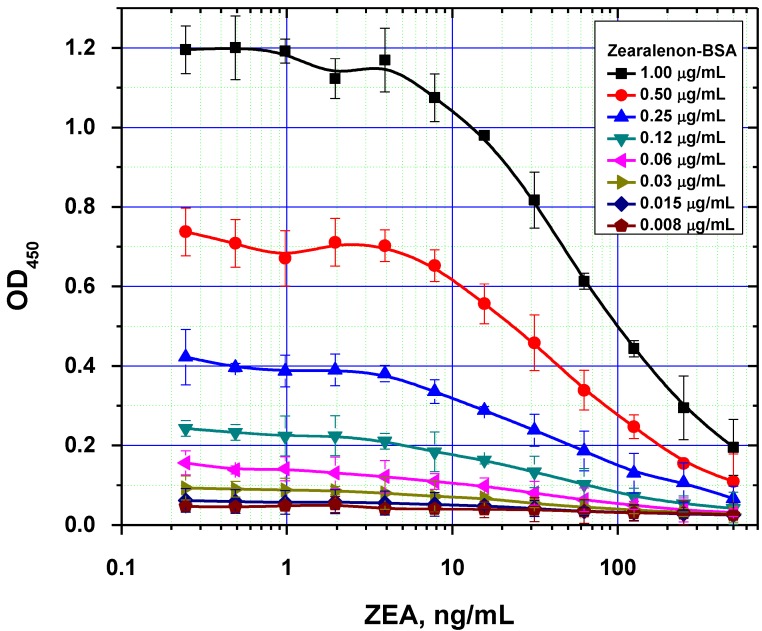
Comparison of ELISA calibration curves for different concentrations of immobilized ZEA–BSA conjugate (data are presented for three replicates).

It was also demonstrated that excessive amounts of the immobilized reagent can result in two opposing effects. On one hand, it contributes to more efficient binding of the antibodies on the microplate surface. On the other hand, this negatively affects the analytical performance by increasing the detection limit, because of higher concentration of the competitors are necessary to inhibit antibody binding with solid phase (Figure 4). As a result, the following values were chosen as optimal concentrations for rapid ELISAs under the equilibrium regime: 0.5, 0.3 and 1.0 µg/mL for AFB1-STI, OTA-BSA and ZEA-BSA, respectively.

### 2.4. Reducing the Duration of the Competitive Immunochemical Interaction

**Figure 5:**
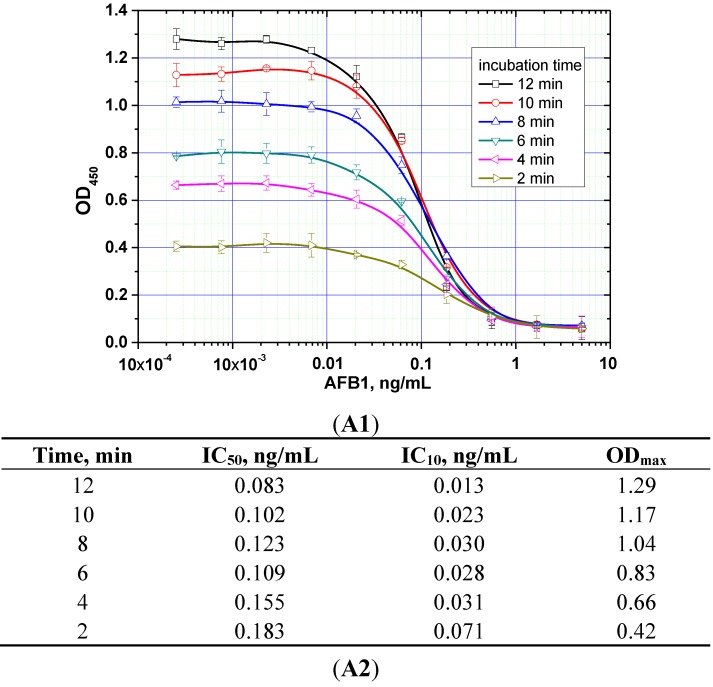

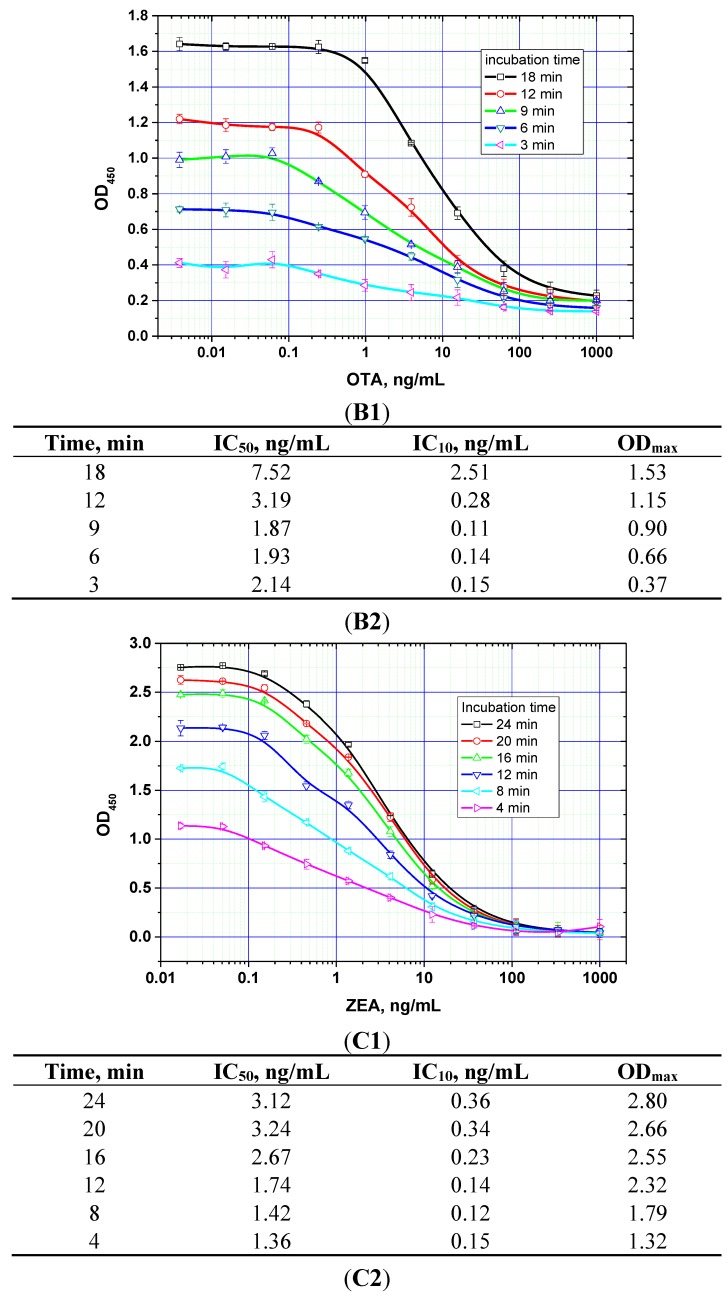
Competitive curves of the immunoenzymatic determination of AFB1 (**A1**), OTA (**B1**), and ZEA (**C1**) and the analytical parameters there of (**A2**, **B2**, and **C2**, respectively) for different durations of the immunochemical interaction (IC_50_ for competitive ELISA is deemed the most accurate point for quantitative measurements, whereas IC_10_ is deemed the limit of detection). Data are presented for three replicates.

The duration of the competitive interaction was varied between 2 and 24 min (further increases in the duration did not result in reproducible increases in the signal amplitude). Examples of the corresponding experiments are shown in Figure 5. For rapid ELISA of ZEA, increasing the duration of the competitive interaction resulted in a lower detection limit. With an incubation period up to 12 min, the detection limit reached <0.1 ng/mL (see Figure 5C). However, even a 4-min incubation produced a signal amplitude >1.0. With a view towards optimized duration, the relevant optimal concentrations of the reagents were then selected for the kinetic ELISA method. Our optimization criterion was the choice of the minimum durations of the stages at which the signal amplitude is large enough (OD > 0.5) for the correct quantitative measurement of the assay results. Comparisons for the other two mycotoxins (see Figure 5A,B) similarly informed the choice of optimal conditions for the competitive stage of ELISA, as summarized in Table 3.

**Table 3:** Selected conditions for the competitive immunochemical interaction and the resulting characteristics of the mycotoxin ELISAs.

Mycotoxins	Concentration of antibodies, ng/mL	Concentration of the mycotoxin–protein conjugate, µg/mL	Duration of the stage, min	Limit of detection, ng/mL	OD_max_
AFB1	120	0.5	8	0.03	0.8
OTA	500	0.4	9	0.13	0.9
ZEA	100	1.0	4	0.15	1.3

### 2.5. Reducing the Duration of the Interaction between the Biotinylated Immune Complexes and the Streptavidin–Polyperoxidase Conjugate

The duration of the second assay step was varied between 3–18 min, while the first step had the relevant fixed duration as previously determined for each mycotoxin (see above). The parameters of the obtained competitive curves are summarized in Table 4.

**Table 4:** Signal amplitude and detection limit achieved using varying incubation periods for the interaction between the biotinylated immune complexes and streptavidin–polyperoxidase conjugate (data are presented for three replicates).

Mycotoxins	Time, min				
	3	6	9	12	18
	Signal amplitude, OD_450_/detection limit, ng/mL				
AFB1	0.45/0.03	0.60/0.02	0.75/0.02	0.78/0.03	0.79/0.03
OTA	0.78/0.14	0.95/0.13	0.94/0.13	0.96/0.14	0.95/0.12
ZEA	0.80/0.21	0.88/0.11	0.92/0.23	1.10/0.30	1.15/0.30

The selection criterion was OD_max_ > 0.5. Accordingly, the duration of this stage for the rapid ELISA was chosen to be 6, 3, and 3 min for AFB1, OTA, and ZEA, respectively. It was also noted that reductions in the incubation time for this step had no effect on the observed detection limits.

### 2.6. Choice of Protocol for Kinetic Multianalysis of Mycotoxins

Once the reagent concentrations used at each step were optimized under the above determined incubation conditions, the assay protocols for kinetic ELISAs for the three individual mycotoxins could be finalized. The obtained results suggested the possibility of carrying out all three assays simultaneously, with a total assay time not exceeding 25 min (see Table 5), and without any significant deterioration in analytical parameters compared with ELISA carried out in equilibrium mode.

**Table 5:** Duration of rapid ELISA stages for the three mycotoxins studied.

Mycotoxin	Competition interaction, min	Interaction with HRP conjugate, min	Enzymatic reaction, min	Total assay time *, min
AFB1	8	6	8	24
ОТА	9	3	8	22
ZEA	4	3	8	17

* Including microplate washes and reagent addition between stages.

Combining these optimized protocols for application towards the simultaneous control of all three mycotoxins resulted in the following assay regime: 8-min duration for the competitive interaction, 6-min incubation with the peroxidase conjugate, and 8-min duration for the enzymatic reaction. This resulted in a total duration of 25 min for the multianalytical assay (including 3 min for auxiliary operations).

Transition to this regime did not lead to a significant change in the immunoassay characteristics of each analyte compared with the individual assays. Thus, a five-fold reduction in the total analysis time was obtainable relative to the equilibrium mode (see Table 1 and Table 6), without any significant deleterious effects on the analytical characteristics, yet with greatly increased speed and throughput.

**Table 6:** Analytical characteristics of mycotoxin ELISA in the simultaneous kinetic regime.

Mycotoxin	Analysis time, min	Limit of detection, ng/mL	Maximum deviation *	Average deviation *
AFB1	25	0.02	14.4%	6.2%
OTA		0.1	14.5%	4.7%
ZEA		0.25	15.0%	6.8%

* Measurements were carried out at four different concentrations within the working range; *n* = 4.

Taking into consideration the extraction steps, the detection limits were equal to 0.24, 1.2 and 3 ng/g and working ranges were 0.25–10, 2–400, 5–500 ng/g for AFB1, OTA, and ZEA respectively.

### 2.7. Application of the Developed Methods for Testing of Real Samples

Raw extracts from poultry processing preparations and corn were taken for the testing of the developed methods. Mycotoxin extraction from was performed using standard procedures and a five-fold excess of a 70:30 methanol-water mixture. It should be noted that the used water-organic extraction is carried out by the same protocol thus allowing subsequent analysis of all three mycotoxins in the same extract.

Firstly, the absence of AFB1, OTA and ZEA in these matrixes was demonstrated using LC/fluorescence, LC/MS or HPLC methods [40], the data were provided by University of Parma, Italy (Prof. A. Dossena) for poultry processing preparations and “Test-Pushchino”, Ltd., Russia (Dr. M. Voznyak) for corn samples. Then the extracts were spiked by known quantities of the mycotoxins. Initial extracts, spiked extracts and pure mycotoxins solutions were compared by the developed ELISA technique.

The calculations of the AFB1 content for spiked extracts based on the ELISA calibration curve give very close values (second column of the Table 7 and Table 8 for the AFB1 experiments) to the known added concentrations of the AFB1 (the first column). Besides, initial (not containing mycotoxins) extracts did not affect the recorded OD in the ELISA. The found value of AFB1 (basing on calibration curve for pure AFB1 solutions) was 0 ng/g for all repetitions.

Similar trends were demonstrated for two other studied mycotoxins (see Table 7 and Table 8 for OTA and ZEA) and confirmed reliable measurements of mycotoxins in real samples by the proposed ELISA techniques. Recovery of the added mycotoxins varied from 94.0% to 111.2%.

The average deviations did not exceed 10%, whereas the average recovery of AFB1, OTA, and ZEA were 98%, 102%, and 101%, respectively.

According to European Commission regulations [41], the maximum permissible levels of AFB1, OTA, and ZEA in foodstuffs are 2, 2, and 20 ng/g respectively. The proposed system therefore provides sufficient assay sensitivity for the purposes of practical application.

**Table 7:** Determination of mycotoxins in extracts of animal product samples.

Added, ng/g	Found *, ng/g	Average deviation of found values, ng/g	Recovery, %
AFB1			
8.4	8.2	0.2	97.6
3.4	3.3	0.2	97.1
1.0	1.0	0.1	100.0
0.3	0.3	0.1	100.0
0	0	0	-
OTA			
120	118.2	0.7	98.5
32	35.5	0.6	111.2
8	7.5	0.1	94.0
2	1.9	0.1	96.0
0	0	0	-
ZEA			
200	198.2	13.6	99.1
70	72.2	7.7	103.1
24	25.4	2.2	105.8
8	7.7	0.5	96.2
0	0	0	-

* Measurements were carried out at four concentrations within the working range and for non-spiked samples; *n* = 5.

**Table 8:** Determination of mycotoxins in extracts of corn.

Added, ng/g	Found *, ng/g	Average deviation of found values, ng/g	Recovery, %
AFB1			
10	10.0	0.1	100.0
5	4.9	0.1	98.0
1	1.0	0.1	100.0
0	0	0	-
OTA			
50	50.2	0.2	100.4
25	24.5	0.3	98.0
10	9.7	0.1	97.0
0	0	0	-
ZEA			
100	104.1	5.1	104.1
50	54.2	4.3	108.4
10	9.6	1.6	96.0
0	0	0	-

* Measurements were carried out at four concentrations within the working range and for non-spiked samples; *n* = 5.

## 3. Experimental Section

### 3.1. Materials

Ochratoxin A, 3,3' 5,5'-tetramethylbenzidine (TMB), Triton X-100, dimethyl sulfoxide, biotin *N*-hydroxysuccinimide ester, biotinamidohexanoy-l-6-aminohexanoic acid *N*-hydroxysuccinimide ester, and the streptavidin-peroxidase polymer were from Sigma-Aldrich (St. Louis, MO, USA; www.sial.com). Aflatoxin B1 and zearalenone were from Chromresurs, Ltd. (Moscow, Russia; www.chromresurs.ru).

Monoclonal antibodies against ochratoxin A (clone K30.88) and zearalenone (clone H1.44) were provided by Petr Georgievich Sveshnikov (Russian Research Center of Molecular Diagnostics and Therapy, Laboratory of Biotechnology, Moscow, Russia; www.hybridoma.ru). OTA conjugated with bovine serum albumin (BSA) and monoclonal antibodies against AFB1 were provided by IL-TEST Pushchino, Ltd. (Pushchino, Moscow, Russia, www.test-p.ru). Zearalenone–BSA and soybean trypsin inhibitor (STI)-AFB1 conjugates were provided by Prof. Sergey Alexandrovich Eremin (Moscow State University, Chemistry Faculty, Department of Chemical Enzymology, Moscow, Russia). Antibody specificities were confirmed previously [34]. Peroxidase-labeled anti-mouse immunoglobulins were obtained from the N.F. Gamaleya Institute of Epidemiology and Microbiology (Moscow, Russia; www.gamaleya.ru). All other reagents were of analytical grade purity or greater.

Deionized water (18 MΩ·cm at 25 °C, Simplicity Millipore, Billerica, MA, USA; www.millipore.com) was used for the preparation of all solutions. ELISA was performed with Costar 9018 microplates (Corning, New York, NY, USA). When conducting ELISA, absorbance of the reaction product was detected with a Zenyth 3100 microplate reader (Anthos Labtec Instruments, Salzburg, Austria).

Functional animal protein (FAP) and corn samples from the poultry industry were used as a model matrix. Corn for the studies was purchased in local market. FAP samples were manufactured by the All-Russian Research Institute for Poultry Processing Industry within the European project PROSPARE (www.prospare.eu). Unmarketable residues (feathers, bones, carcasses) were processed by enzyme blends at 55 °C under the optimized conditions providing 70%–80% of soluble peptide product with average molecular weight 6.3 kDa, which is subsequently used as a recycled component for food stuffs manufacturing [42].

Extraction of mycotoxins from FAP and corn samples was performed as previously described [43], with some modifications. Solid substance (FAP, milled grains) was mixed with an extraction solution (70% methanol, 30% water) at a ratio of 1:5, and incubated with gentle stirring at room temperature.

### 3.2. Biotinylation of Antibodies

Biotinylation was performed using standard protocols [44]. Solutions of antibodies against OTA, AFB1 and ZEA were prepared at a 3-mg/mL concentration in 2× PBS. *N*-hydroxysuccinimidyl ester of biotin or *N*-hydroxysuccinimide ester of biotinamidohexanoyl-6-aminohexanoic acid [35,36] were dissolved in dimethylsulfoxide (10 mg/mL) and added to the antibodies at a 20-fold molar excess. After 2 h incubation at room temperature with vigorous stirring, unreacted low molecular components were separated by dialysis three times against PBS.

### 3.3. ELISA of Mycotoxins

AFB1-STI, OTA-BSA or ZEA-BSA were incubated in separate wells of a microplate overnight at 4 °C at concentrations of 0.5 μg/mL in PBS. After four washes with PBS containing 0.05% Triton X-100 (PBST), 100 uL of a mixture of specific antibodies at a concentration of 0.1–0.5 mg/mL and antigen-containing samples at a concentration of 0.001–1.000 ng/mL (in PBST or 3:1 PBST:methanol mixture) were added into the wells and incubated for 2–60 min at 37 °C. The same washing procedure was performed, after which a PBST solution of diluted peroxidase-labeled anti-mouse immunoglobulins (1:6000), avidin–peroxidase conjugate (1:12,000) or streptavidin–polyperoxidase conjugate (1:4000), were added at 100 µL per well and incubated for 4–60 min at 37 °C. The microplate wells were then washed with PBST. To determine the peroxidase activity, the substrate solution (0.42 mM TMB and 1.8 mM H_2_O_2_ in 0.1 M sodium citrate buffer, pH 4.0; 100 µL per well) was injected. After incubation at room temperature for 3–15 min, the reaction was terminated by the addition of 100 µL of 1 M H_2_SO_4_. The absorbance of the reaction product was read at 450 nm.

Dependences of the integrated coloration (y) on the concentration of the antigen in the sample (x) were estimated using the Origin 7.5 software package (Origin Lab, Northampton, MA, USA; 2003, www.originlab.com) by employing the four-parameter sigmoid function Equation (1),

(1) $$y=\frac{A_{1}-A_{2}}{1+{\left(\frac{x}{c}\right)}^{b}}+A_{2}$$

where A1 is the maximum value of the signal, *A2* is the minimum value of the signal, *c* is the antigen concentration that inhibits binding of the antibodies to the immobilized antigen by 50% (IC_50_), and b is the slope of the curve at IC_50_ [45]. This function was used to determine the antigen limit of detection, which corresponds to 10% inhibition of binding, *i.e.*, IC_10_ [46].

### 3.4. ELISA of Mycotoxin-Containing Extracts

The mycotoxin–protein conjugates were adsorbed in separate microplate wells and washed as described above. Samples in solution (50 µL) with a methanol content of 25%, and 50 µL of specific antibodies in PBST (at concentrations of 100, 100, and 500 ng/mL for AFB1, OTA, and ZEA, respectively) were added into the wells and incubated for 8 min with vigorous stirring at room temperature. After washing, a diluted solution of the streptavidin–polyperoxidase conjugate (1:4000 in PBST) was added at 100 µL per well and incubated for 6 min at 37 °C with vigorous stirring. The microplate was then washed four times with PBST, and following an 8 min incubation with the substrate solution the formed immune complexes were detected and quantitatively characterized as described above.

## 4. Conclusions

We have developed and validated an approach for multiplex ELISA in kinetic mode. The application of streptavidin–polyperoxidase and a biotin-modified antibody with an elongated spacer was combined with step-by-step reduction of the duration of the assay stages. This assay allows the monitoring and control of mycotoxins aflatoxin B1, ochratoxin A, and zearalenone simultaneously with a total assay duration of 25 min and with detection limits of 0.02, 0.10 and 0.25 ng/mL, respectively. The assay was successfully validated using extracts from poultry-processing functional animal protein preparations and from corn with the detection limits 0.24, 1.2 and 3 ng/g, respectively. Ease of implementation and flexibility of the proposed approach will facilitate its transfer to immunoassays of other relevant substances.
