# Addendum: Urusov, A.E.; Zherdev, A.V.; Petrakova, A.V.; Sadykhov, E.G.; Koroleva, O.V.; Dzantiev, B.B. Rapid Multiple Immunoenzyme Assay of Mycotoxins. *Toxins*, 2015, *7*, 238-254. 

**DOI:** 10.3390/toxins7062134

**Published:** 2015-06-11

**Authors:** Alexandr E. Urusov, Anatoly V. Zherdev, Alina V. Petrakova, Elchin G. Sadykhov, Olga V. Koroleva, Boris B. Dzantiev

**Affiliations:** A.N. Bach Institute of Biochemistry of the Russian Academy of Sciences, Leninsky Prospect 33, 119071 Moscow, Russia; E-Mails: urusov.alexandr@gmail.com (A.E.U.); zherdev@inbi.ras.ru (A.V.Z.); alina.petrakova@gmail.com (A.V.P.); elchins@gmail.com (E.G.S.); koroleva@inbi.ras.ru (O.V.K.)

The authors would like to replace the Acknowledgements section with the following:

**“Acknowledgements**

This work was supported by the Russian State Targeted Program «Research and Development in Priority Areas of Development of the Russian Scientific and Technological Complex for 2014–2020» (contract 14.607.21.0015 from 5 June 2014; unique identifier of applied research: RFMEFI60714X0015), Russian Foundation for Basic Research (grant 14-03-00753).”

